# Evidence of Drought Stress Memory in the Facultative CAM, *Aptenia cordifolia*: Possible Role of Phytohormones

**DOI:** 10.1371/journal.pone.0135391

**Published:** 2015-08-14

**Authors:** Eva Fleta-Soriano, Marta Pintó-Marijuan, Sergi Munné-Bosch

**Affiliations:** Departament de Biologia Vegetal, Facultat de Biologia, Universitat de Barcelona, Avinguda Diagonal, 643, E-08028, Barcelona, Spain; University of Manitoba, CANADA

## Abstract

Although plant responses to drought stress have been studied in detail in several plant species, including CAM plants, the occurrence of stress memory and possible mechanisms for its regulation are still very poorly understood. In an attempt to better understand the occurrence and possible mechanisms of regulation of stress memory in plants, we measured the concentrations of phytohormones in *Aptenia cordifolia* exposed to reiterated drought, together with various stress indicators, including leaf water contents, photosynthesis and mechanisms of photo- and antioxidant protection. Results showed that plants exposed to drought stress responded differently if previously challenged with a first drought. Gibberellin levels decreased upon exposure to the first drought and remained lower in double-stressed plants compared with those exposed to stress for the first time. In contrast, abscisic acid levels were higher in double- than single-stressed plants. This occurred in parallel with alterations in hydroperoxide levels, but not with malondialdehyde levels, thus suggesting an increased oxidation state that did not result in oxidative damage in double-stressed plants. It is concluded that (i) drought stress memory occurs in double-stressed *A*. *cordifolia* plants, (ii) both gibberellins and abscisic acid may play a role in plant response to repeated periods of drought, and (iii) changes in abscisic acid levels in double-stressed plants may have a positive effect by modulating changes in the cellular redox state with a role in signalling, rather than cause oxidative damage to the cell.

## Introduction

Stress memory in plants, also known as stress imprint or priming, is considered to be an important component of the behavioral ecology of plants and it is becoming an increasingly important part of plant stress physiology textbooks nowadays. Stress memory is defined as the capacity of organisms to respond better to a given stress factor when individuals have already been challenged previously with the same stimulus relative to those that have not been exposed to the stress before [1]. Indeed, there is an increasing interest in stress memory effects, since this feature has important implications in plant stress physiology [2]. Unfortunately, the occurrence of stress memory, either leading to positive or negative effects in plant stress responses, and the mechanisms underlying stress memory are still very poorly understood [2]. Therefore, a better knowledge on stress memory effects is urgently needed not only to better understand the physiology and ecology of plants, but also to improve crop production and environmental management practices.

Mechanisms of stress memory may largely vary depending on the organizational level to what the studies are carried out, from changes in leaf anatomy to epigenomics, including phenological, biochemical and physiological mechanisms that may operate in an integrated way to fulfill a role in plant stress tolerance. Among these mechanisms, it appears that phytohormones may have a prominent role. Gibberellins (GAs) have long been known to be involved in vernalization, which implies epigenetic changes and long-term memory effects [3]. On the other hand, recent studies suggest that abscisic acid (ABA) may be involved in short-term drought stress acclimation in the model plant, *Arabidopsis thaliana*. It has been shown that *A*. *thaliana* increase the transcription of several ABA-induced genes in response to reiterated dehydration, while maintaining leaf water contents [4,5]. Guard cells appear to have a dehydration stress memory so that plants produce ABA to keep partially closed stomata in order to reduce water loss under reiterated water deficit conditions [6].

Crassulacean acid metabolism (CAM) is an adaptive mechanism to survive in extreme habitats characterized by severe drought in which the carbon dioxide is assimilated during the night avoiding an excessive water loss [7]. In fact, CAM plants are able to keep a minimal metabolically active state for a long time during severe droughts, while they are able to recover quickly during re-watering [8]. Therefore, drought stress memory in CAM plants, which are specialized in drought stress tolerance, may have a tremendous biological significance. While drought stress responses have been extensively studied in CAM plants [9–11], the occurrence of stress memory in CAM plants and the possible mechanisms for its regulation are still very poorly understood.

Here, we hypothesized that the CAM plant, *Aptenia cordifolia* may show a drought stress memory in plant response to drought stress, in which phytohormones could play a role. Since *A*. *cordifolia* is an invasive species, this capacity could help displace other species less resistant to drought stress and colonize new habitats. Specifically, in the present study, we examined whether or not *A*. *cordifolia* show any stress memory to reiterated drought. With this aim, we measured the endogenous levels of phytohormones, together with various markers of physiological stress, in double- compared with single-stressed plants.

## Materials and Methods

### Plant material, treatments and sampling

Sixty plants of baby sun rose (*Aptenia cordifolia* (L.f.) Schwantes) were purchased in a local garden (Ca L’Agustí, Barcelona, Spain) and were transferred to 0.5 L-pots with peat:perlite:vermiculite (2:1:1, v/v). Plants were grown in a greenhouse at the Faculty of Biology of the University of Barcelona (Barcelona, Spain). Prior to experiments, plants were watered 3 times per week with half-diluted Hoagland nutrient solution. Experiments, which started on 11th June 2014, consisted in developing two water regimes on plants: CS plants were watered for 13 days, exposed to drought by withholding water for 10 days, and then re-watered for 4 days; while SS plants were stressed by withholding water for 9 days, recovered for 4 days and then exposed again to drought for 10 days, followed by a final recovery of 4 days. Therefore, SS were double-stressed, while CS plants were stressed during a single period. Samplings were performed at the beginning of the experiment (day 0) and after 9, 13, 23 and 27 days of treatments, that is at the points of maximum stress and during recovery. All measurements were performed at midday (between 11 and 13h solar time). At each sampling point, fully-expanded young leaves of 7 individuals were used to estimate the endogenous contents of phytohormones, together with various physiological indicators, including leaf water contents and gas exchange, chlorophyll fluorescence, levels of photosynthetic pigments and antioxidants, and the extent of lipid peroxidation in leaves. Samples for phytohormone and other biochemical analyses were collected, immediately frozen in liquid nitrogen and stored at -80°C until analysis.

### Hormonal profiling

The extraction and analysis of GAs, including the bioactive GA_1_ and GA_4_, and their precursors GA_9_, GA_19_, GA_20_ and GA_24_, the bioactive auxin, indole-3-acetic acid (IAA), the cytokinins, zeatin (Z), zeatin riboside (ZR), 2-isopentenyl adenine (2iP) and isopentenyl adenosine (iPA), and the stress-related phytohormones, ABA, jasmonic acid (JA) and salicylic acid (SA) were carried out by UPLC-MS/MS as described [12]. Deuterium-labelled phytohormones were used as internal standards.

### Leaf water contents, gas exchange and chlorophyll fluorescence

Samples were weighed to estimate the fresh matter (FW), immersed in distilled water at 4°C for 24h to estimate the turgid matter (TW) and then oven-dried at 80°C to constant weight to estimate the dry matter (DW). Relative water content (RWC) was then calculated as 100x(FW-DW)/(TW-DW). Net photosynthesis (A), stomatal conductance (g_s_) and the maximum efficiency of photosystem II phptochemistry (*F*_v_/*F*_m_) were estimated by using a portable infrared gas analyzer with a leaf chamber fluorometer (LI-COR 6400 system, LI-COR, Lincoln, NE, USA). Light intensity was set at 700 μmol quanta m^-2^ s^-1^ with a 10% of blue light; CO_2_ concentration at 400 ppm; leaf temperature at 20–25°C, and relative humidity ranging 50–60% with a flow of 500 μmol s^-1^. The *F*_v_/*F*_m_ ratio, which was measured after adapting the leaves to darkness for 2 h, was calculated as (*F*_m_-*F*_0_)/ *F*_m_, where *F*_m_ and *F*_0_ are the maximum and basal fluorescence yields, respectively, of dark-adapted leaves [13].

### Photosynthetic pigments and antioxidants

For pigment and tocopherol analysis, leaf samples (50 mg) were ground in liquid nitrogen and extracted with cold methanol (v/v) using ultrasonication. After centrifuging at 8000 rpm for 10 min and 4°C, the supernatant was collected and the pellet was re-extracted with the same solvent until it was colourless. Then, supernatants were pooled and filtered through a 0.5 μm syringe filter. Total chlorophylls and carotenoids were estimated spectrophotometrically as described [14]. Levels of neoxanthin and violaxanthin, ABA precursors, were measured by high performance liquid chromatography (HPLC) as described [15]. Tocopherols were measured by HPLC as described [16].

### Estimation of lipid peroxidation

The extent of lipid peroxidation was estimated by measuring the levels of lipid hydroperoxides (primary stable products of lipid peroxidation) and malondialdehyde (MDA) equivalents (secondary products of lipid peroxidation) in leaves. Lipid hydroperoxides levels were estimated spectrophotometrically following the ferrous oxidation-xylenol orange assay as described [17]. MDA levels were estimated spectrophotometrically following the thiobarbituric acid-reactive assay considering the effect of potential interfering compounds, as described [18].

### Statistical analysis

In the first set of results, which included a time-course evolution of water contents, leaf gas exchange and phytohormone levels, data was analyzed by using two-way factorial analysis of variance (ANOVA) with treatment and time (sampling day) as factors, and by additionally using Duncan posthoc tests. In the second set of results, in which differences in photosynthetic pigments, antioxidants and the extent of lipid peroxidation in a single time point were analyzed, Student´s t-tests were used. In all cases, differences were considered significant at a probability level of *P*<0.05. All statistical tests were carried out using the SPSS 15.0 statistical package (SPSS, Inc., Chicago, IL, USA).

## Results and Discussion

### Drought-stressed *A*. *cordifolia* plants induce CAM metabolism

**Fig 1:**
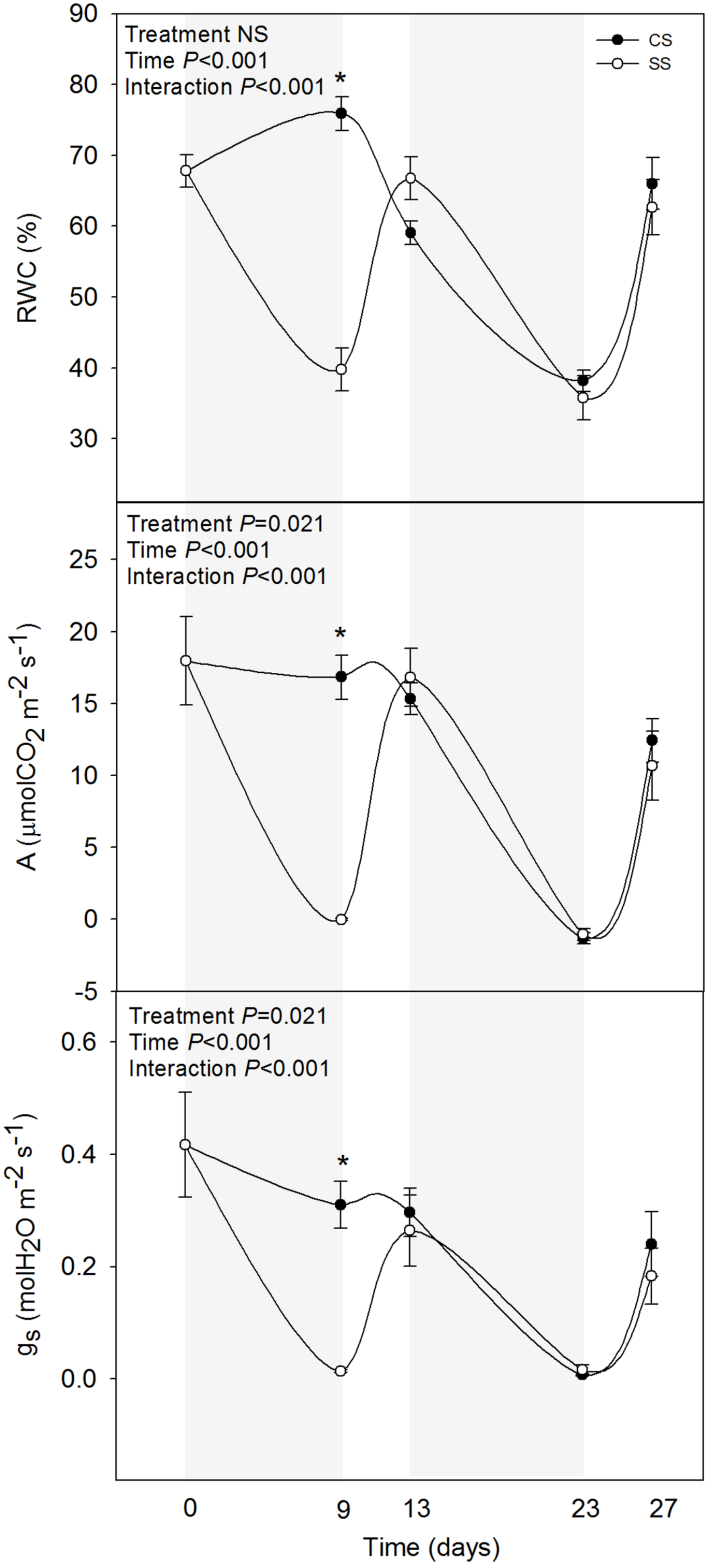
Relative water content (RWC), CO_2_ assimilation (A) and stomatal conductance (g_s_) in leaves of *A*. *cordifolia*. Data represent the mean ± SE of *n* = 7 individuals. Significant differences between groups were tested by two-way analysis of variance (ANOVA) and Duncan posthoc tests.

A number of CAM plants are extremely resistant to drought, as they are commonly adapted to deserts and other arid or semi-arid environments [19]. Aside from stomatal closure during the day, which largely increases water use efficiency, CAM plants adopt a series of strategies to resist water deprivation during long periods, including, among others, specific hormonal responses and activation of mechanisms of photo- and antioxidant protection [9]. *A*. *cordifolia* was first described as a facultative CAM species [20], and later thought to be an obligate CAM [21]. In the present study, however, it is shown that it is a facultative CAM plant that opens stomata at midday when water is available and closing them completely when water is withheld (Fig 1). After 9 days of withholding water, the RWC decreased from 68% to 40% at midday. This was associated with a sharp reduction in CO_2_ assimilation and stomatal conductance rates, which reached values of and close to zero, respectively (Fig 1). The stress caused to plants, as indicated by RWC values, was quite severe. When challenged with a new drought, plants reduced again the RWC and gas exchange values to a similar extent. Double-stressed plants did not respond differently as those stressed for the first time in terms of leaf water contents and gas exchange (Fig 1), thus indicating that a second stress did impact neither positively nor negatively on these parameters.

### GAs and ABA levels reveal stress memory

**Fig 2:**
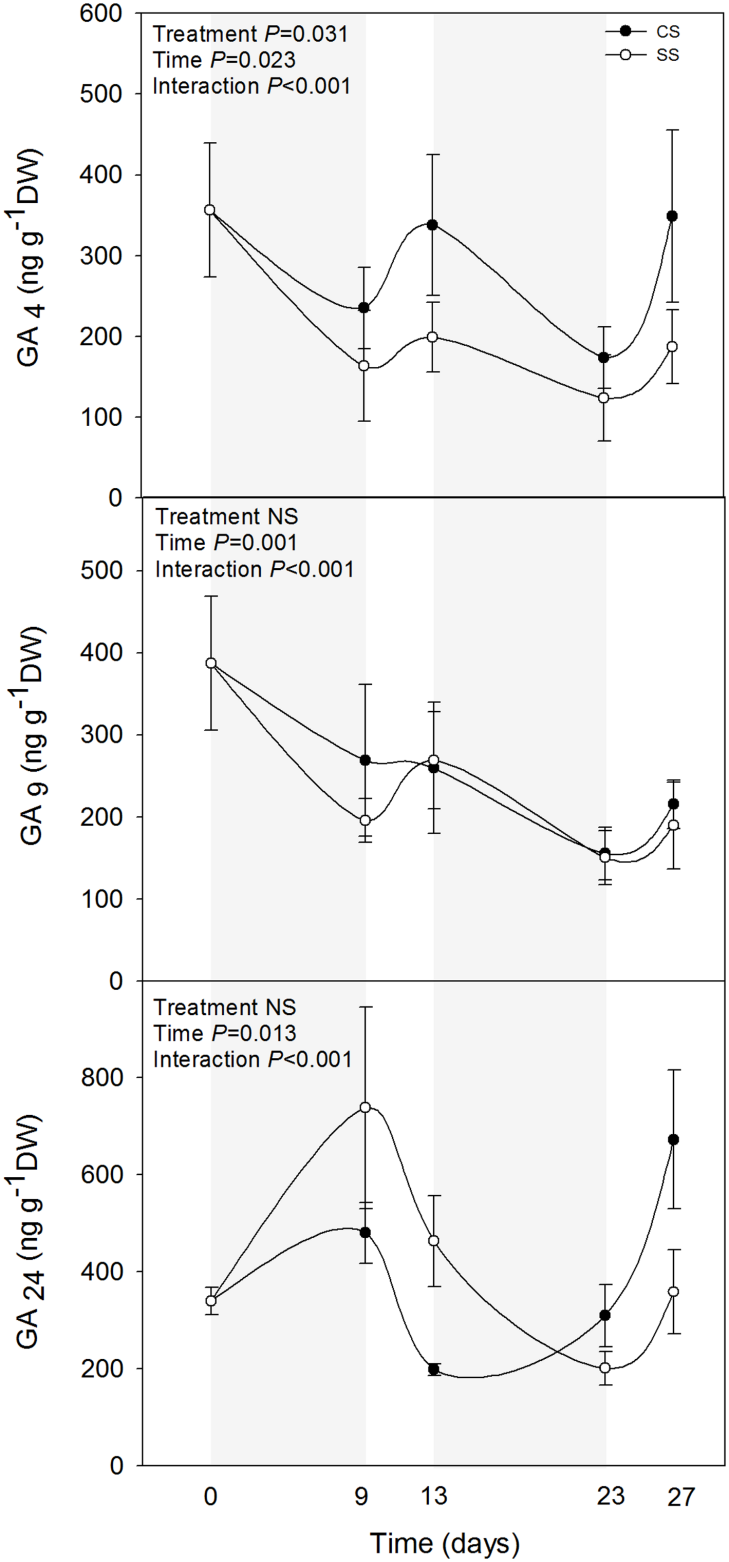
Endogenous concentrations of gibberellin 4 (GA_4_), and its precursors, gibberellin 9 (GA_9_) and gibberellin 24 (GA_24_) in leaves of *A*. *cordifolia*. Data represent the mean ± SE of *n* = 7 individuals. Significant differences between groups were tested by two-way analysis of variance (ANOVA) and Duncan posthoc tests.

**Fig 3:**
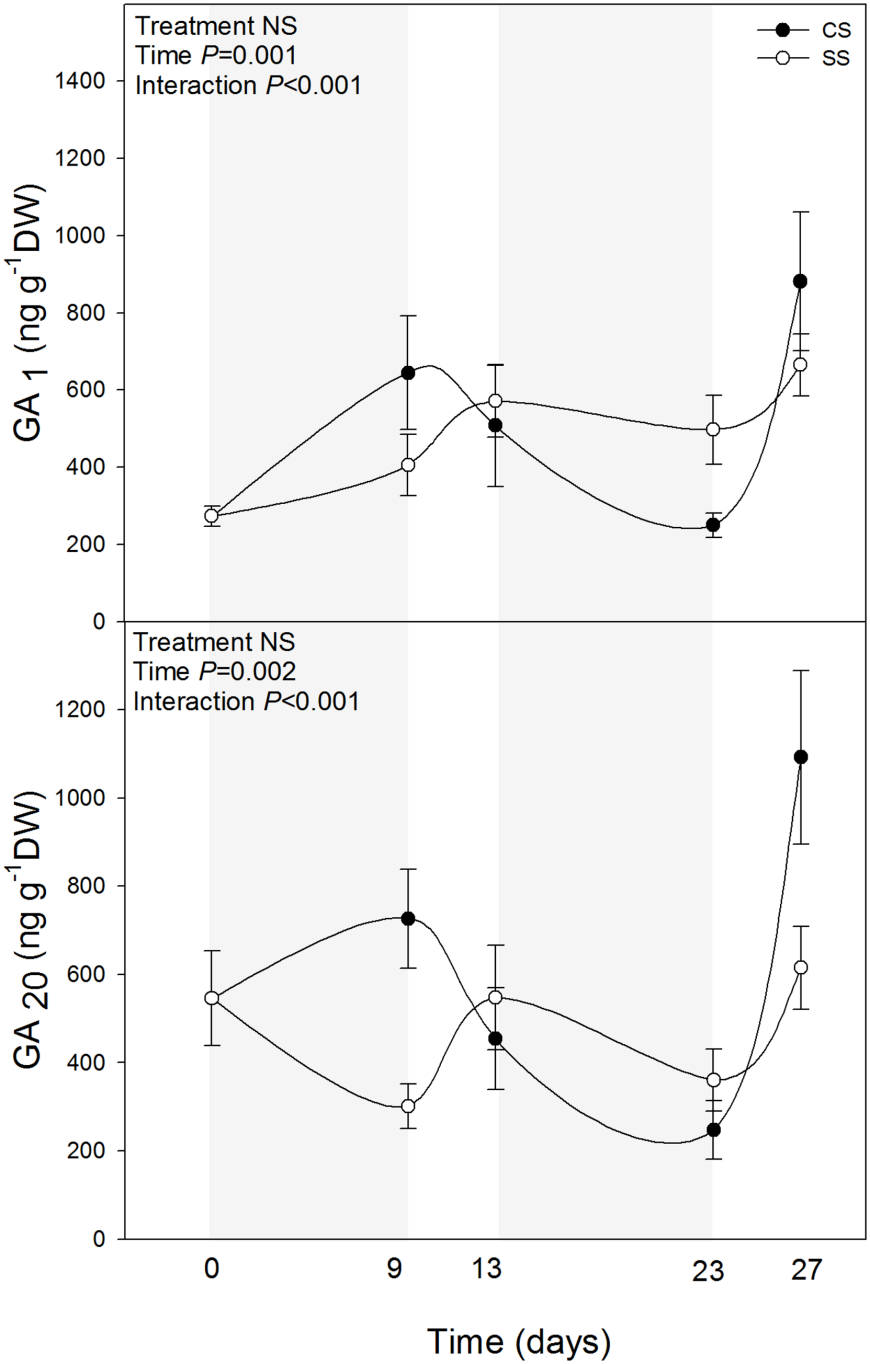
Endogenous concentrations of gibberellin 1 (GA_1_), and its precursor, gibberellin 20 (GA_20_) in leaves of *A*. *cordifolia*. Data represent the mean ± SE of *n* = 7 individuals. Significant differences between groups were tested by two-way analysis of variance (ANOVA) and Duncan posthoc tests.

Among the analysed phytohormones, the bioactive GA_4_ was the one showing the most relevant results (Figs 2 and 3, Table 1). GA_4_ levels decreased during drought and did not recover, so that double-stressed plants showed slightly, but consistently lower GA_4_ levels throughout the experiment compared with plants challenged by drought for the first time (Fig 2). While GA_9_ levels were not affected by drought stress, the endogenous concentrations of GA_24_ increased during the first drought, to decrease later during the second drought in double-stressed plants (Fig 2). Since GA_24_ is a precursor of GA_4_ [22], it is likely that conversion to bioactive GAs is reduced during the first drought, thus leading to an accumulation of GA precursors. When challenged again with a second stress, however, it seems that this effect disappears, so that GA precursors do not accumulate (despite bioactive GA levels were kept at low levels). Although still to be confirmed using enzymatic assays and molecular tools, these results suggest a memory effect on GA metabolism in plant response to reiterated drought, in analogy to the regulation of GA metabolism by vernalization [23]. It is noteworthy that levels of GAs from the GA_4_ pathway were affected by reiterated stress (Figs 2 and 3), and that neither auxin, cytokinins, salicylic acid nor jasmonic acid levels were affected by repeated periods of drought (Table 1).

**Table 1:** *P* values of the analysis of variance (ANOVA) to test the effect of treatment, sampling time and its interaction on the levels of phytohormones in leaves of *A*. *cordifolia*.

Hormone	Treatment	Time	Interaction
IAA	NS	0.001	0.003
iPA	NS	0.001	NS
2iP	NS	0.001	0.008
Z	NS	0.041	NS
ZR	NS	0.001	0.001
SA	NS	NS	0.001
JA	NS	0.042	0.001

IAA, indole-3-acetic acid; iPA, isopentenyl adenosine; 2iP, isopentenyl adenine; Z, zeatin; ZR, zeatin riboside; SA, salicylic acid; JA, jasmonic acid. NS, not significant (*P*>0.050).

**Fig 4:**
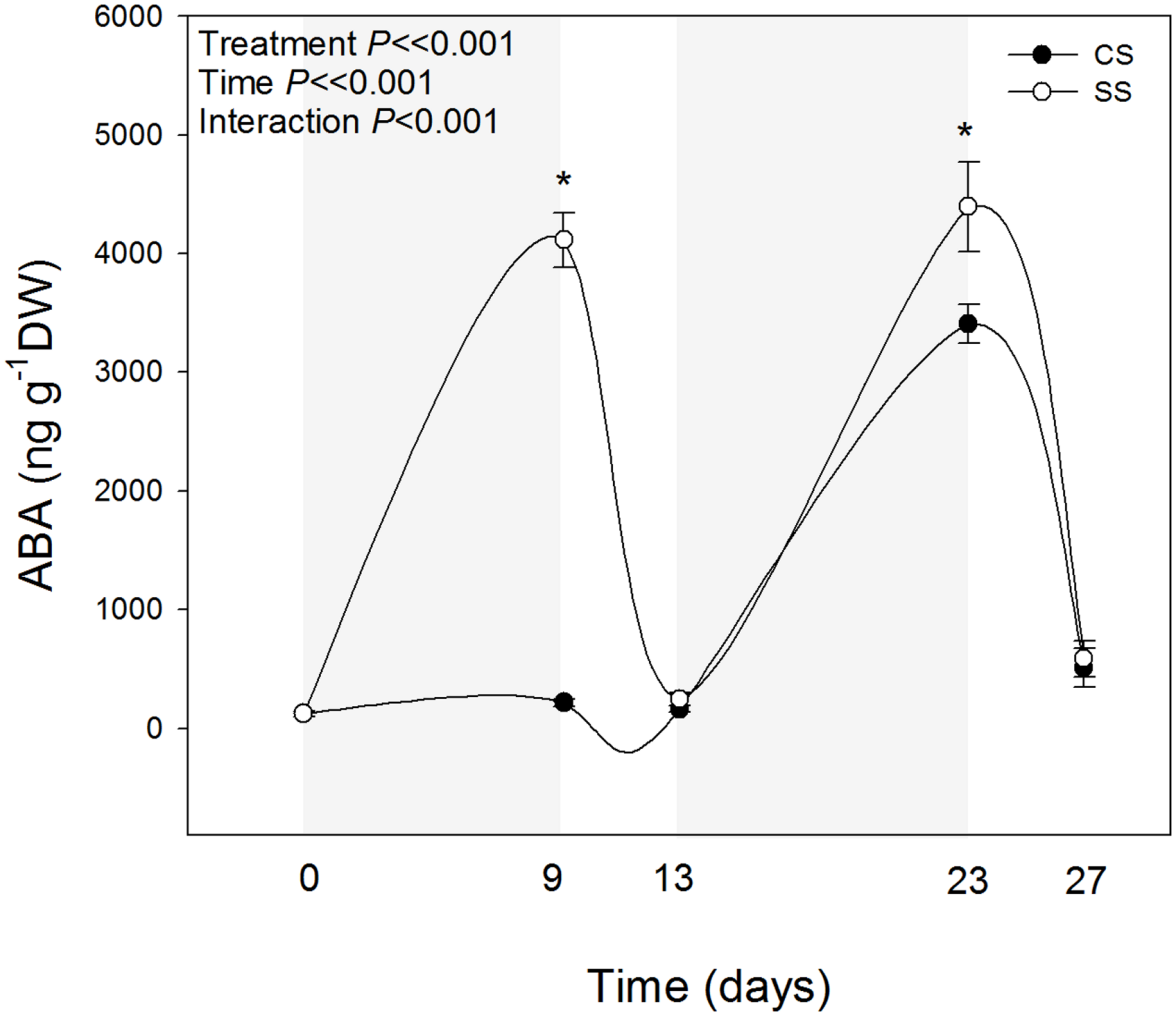
Endogenous concentrations of abscisic acid (ABA) in leaves of *A*. *cordifolia*. Data represent the mean ± SE of *n* = 7 individuals. Significant differences between groups were tested by two-way analysis of variance (ANOVA) and Duncan posthoc tests.

ABA showed differences in double-stressed plants compared with plants challenged with drought for the first time (Fig 4). ABA levels increased 40-fold in response to the first drought to recover later to initial pre-drought values. During the second period of stress, however, ABA levels increased more in double-stressed plants compared with plants challenged by drought for the first time (ABA values reaching 4.2 *vs*. 3.4 μg/g DW, respectively, Fig 4). Despite double-stressed plants did not respond differently in terms of leaf water contents and gas exchange (Fig 1), they did so in terms of ABA accumulation, enhancing the endogenous levels of this phytohormone during the second stress compared with plants challenged by drought for the first time. Increases of ABA levels under reiterated stress may have an effect of growth regulation [24], osmotic adjustment [25] and antioxidant responses [26–28]. Furthermore, previous studies have shown that ABA exerts a protective role under reiterated drought by reprogramming gene expression [4–6]. In the present study, plants did not suffer photo-inhibitory damage to the photosynthetic apparatus, as indicated by constant *F*_v_/*F*_m_ values (Table 2), and plants were able to fully recover after 4 days of re-watering (Fig 1), thus indicating that the observed changes in the endogenous levels of phytohormones may indeed have a positive effect on the physiology of double-stressed plants.

**Table 2:** *F*_v_/*F*_m_ ratio, photosynthetic pigment and antioxidant levels, and extent of lipid peroxidation in double-stressed plants (SS) compared with plants exposed to drought for the first time (CS). Data represent the mean ± SE of *n* = 7.

Parameter	CS	SS
*F*_v_/*F*_m_	0.68±0.02	0.68±0.01
Chlorophyll *a*+*b* (mg/gDW)	11.4±1.0	10.6±0.3
Chlorophyll *a*/*b* (g/g)	1.98±0.01	2.04±0.01*
Carotenoids/ Chlorophyll *a*+*b* (mg/g)	206±2	207±2
Neoxanthin/Chlorophyll *a*+*b* (mg/g)	12.5±1.4	12.0±1.6
Violaxanthin/Chlorophyll *a*+*b* (mg/g)	27.3±0.2	26.3±0.4*
α-Tocopherol/Chlorophyll *a*+*b* (mg/g)	21.9±3.5	23.8±2.0
γ-Tocopherol/ Chlorophyll *a*+*b* (mg/g)	7.3±1.1	6.5±0.5
Ascorbate (μmol/gDW)	11.41±0.67	10.3±0.58
Dehydroascorbate/Total ascorbate (%)	12.43±1.95	11.3±1.95
Lipid hydroperoxides (μmol equiv. H_2_O_2_/gDW)	3.9±0.4	7.4±0.8*
MDA (nmol equiv./gDW)	9.6±2.7	13.6±2.9

* Significant differences between treatments (Student's *t*-test, P<0.050)

ABA could exert a drought memory effect by modulating antioxidant responses. By comparing various photo-oxidative stress and lipid peroxidation markers in double- *vs*. single-stressed plants, it was found that reiterated stress had a significant impact on the chlorophyll a/b ratio and the extent of lipid peroxidation in leaves (Table 2). The chlorophyll a/b ratio increased, however, by 3% only in double- compared with single-stressed plants. In contrast, the extent of lipid peroxidation increased by 90% in double- *vs*. single-stressed plants, as indicated by lipid hydroperoxide levels, respectively. In contrast, malondialdehyde levels were not affected by reiterated drought (Table 2). Lipid hydroperoxide and malondialdehyde levels are the primary and secondary stable products of lipid peroxidation, respectively [29]. Therefore, results obtained suggest that double-stressed plants experienced an increased oxidative stress compared with plants challenged by drought for the first time. However, neither malondialdehyde levels nor the *F*_v_/*F*_m_ ratio were affected by reiterated drought, thus indicating absence of increased photo-oxidative damage in double- *vs*. single-stressed plants [30]. Results obtained appear therefore to be consistent with a hormonal and redox-related gene reprogramming, an aspect that warrants further research. In this respect, it has been previously shown that ABA may trigger activation of antioxidant defences, including vitamin E biosynthesis [31,32]. Despite neither α- nor γ-tocopherol increased in double- *vs*. single-stressed plants (Table 2), a correlative analysis revealed that ABA concentrations positively correlated with γ-tocopherol levels (Table 3). A relationship between ABA and vitamin E biosynthesis is also supported by previous correlative studies in the same species [9] and by studies on model plants, showing that tocopherol-biosynthesis genes have ABA-response elements in their promoter region [33]. Furthermore, analysis of ABA precursors, the carotenoids neoxanthin and violaxanthin, revealed a reduction by 3.7% in violaxanthin levels in double- compared with single-stressed plants (Table 2). Although this reduction appears to be small, it should be considered that it is in the order of 1 mg/g chlorophyll (equivalent to 1.5 mmol/mol chlorophyll), which corresponds to 350 nmol/g DW, while the observed enhanced ABA levels in double- compared with single-stressed plants were in the order of 0.8 μg/g DW (Fig 4), which corresponds to 3 nmol/g DW. Therefore, a 3.7% reduction in violaxanthin levels was more than sufficiently enough to account for the 24% increase of ABA levels in double- compared with single-stressed plants. Further research is however needed to confirm a memory effect on ABA metabolism by directly measuring gene expression, amounts and/or activity of 9-*cis*-epoxycarotenoid dioxygenases, the committed step in ABA biosynthesis from carotenoids [34]. Moreover, it is still to be demonstrated whether or not ABA catabolism is additionally affected in double- compared with single-stressed plants.

**Table 3:** Correlation coefficients and *P* values (in parentheses) of the Spearman rank's correlations between the endogenous concentrations of all phytohormones analyzed and the levels of α- and γ-tocopherol in *A*. *cordifolia* leaves. Significant correlations are indicated in bold (Bonferroni corrected, *P*<0.004).

Hormone	α-Tocopherol	γ-Tocopherol
GA_1_	-0.010 (0.470)	-0.177 (0.107)
GA_4_	-0.286 (0.012)	-0.316 (0.011)
GA_9_	0.001 (0.497)	-0.160 (0.111)
GA_20_	0.141 (0.138)	-0.066 (0.32)
GA_24_	0.157 (0.112)	-0.128 (0.191)
IAA	0.057 (0.331)	-0.217 (0.045)
Z	0.048 (0.357)	-0.262 (0.020)
ZR	0.046 (0.362)	0.072 (0.290)
2iP	0.279 (0.015)	0.285 (0.012)
IPA	-0.223 (0.042)	-0.272 (0.018)
ABA	0.331 (0.005)	**0.552 (<0.001)**
JA	-0.125 (0.167)	-0.218 (0.044)
SA	-0.224 (0.040)	**-0.374 (0.001)**

GAs, gibberellins; IAA, indole-3-acetic acid; Z, zeatin; ZR, zeatin riboside; 2iP, isopentenyl adenine; iPA, isopentenyl adenosine; SA, salicylic acid; JA, jasmonic acid.

## Conclusions

Phytohormones may be involved in plant response to reiterated drought stress in the invasive CAM plant, *A*. *cordifolia*. GA levels decreased upon exposure to the first drought and remained lower in double- compared with single-stressed plants. In contrast, ABA levels were higher in double- than in single-stressed plants. This occurred in parallel with alterations in primary products of lipid peroxidation, but not with changes in malondialdehyde levels and the *F*_v_/*F*_m_ ratio, thus suggesting an increased oxidative stress that did not result in photo-oxidative damage in double-stressed plants. It is concluded that (i) drought stress memory occurs in double-stressed *A*. *cordifolia* plants, (ii) both GAs and ABA may play a role in plant response to repeated periods of drought, and (iii) changes in ABA levels in double-stressed plants may have a positive effect by modulating changes in the cellular redox state with a role in signalling, rather than cause oxidative damage to the cell. In other words, changes in ABA levels in double-stressed plants may be associated with an increased oxidative stress that did not result in photo-oxidative damage. Further research is needed to better understand the GA-, ABA- and redox-mediated effects in plant stress acclimation to reiterated drought in plants.
